# Subcutaneous extraskeletal osteosarcoma of the gluteal region: a case report from a resource-limited setting

**DOI:** 10.1186/s12891-026-10104-8

**Published:** 2026-06-19

**Authors:** Yan Feng, Aleki Fuimaono, Sione Pifeleti, Seventeen Toumoua, Tieying Zhang, Yan Liang, Xu Ding

**Affiliations:** 1 Department of Radiology, Jilin Province People’s Hospital, Changchun, China; 2 Department of Surgery, Tupua Tamasese Meaole Hospital, Apia, Samoa; 3 Department of Pathology, Tupua Tamasese Meaole Hospital, Apia, Samoa; 4 Department of Oncology, Jilin Province People’s Hospital, Changchun, Jilin, China

**Keywords:** Extraskeletal osteosarcoma, Subcutaneous tumor, Soft tissue sarcoma, SATB2, Case report

## Abstract

**Background:**

Extraskeletal osteosarcoma (ESOS) is a rare high-grade malignant mesenchymal tumor that produces osteoid in soft tissue without skeletal involvement. Subcutaneous ESOS is exceptionally uncommon and may mimic benign soft tissue lesions.

**Case presentation:**

A 45-year-old Samoan man presented with a one-year history of a progressively enlarging, painless left gluteal mass. Contrast-enhanced CT showed a well-circumscribed subcutaneous lesion measuring 9.1 × 10.4 × 8.6 cm, with mild to moderate delayed enhancement and no osseous involvement. The tumor was surgically excised. Histopathology showed a spindle to pleomorphic high-grade sarcoma with osteoid produced by atypical tumor cells. Immunohistochemistry showed SATB2 positivity and negative staining for S100, SOX10, pan-cytokeratin, and ERG. The tumor involved the deep resection margin, consistent with R1 resection; the closest margin distance was not specified. The patient declined adjuvant therapy and was subsequently lost to follow-up.

**Conclusions:**

Subcutaneous ESOS is rare and diagnostically challenging. This case highlights the importance of imaging, histopathology, immunohistochemistry, and multidisciplinary management, particularly in resource-limited settings.

## Introduction

Extraskeletal osteosarcoma (ESOS) is a rare, high-grade malignant mesenchymal neoplasm characterized by the production of osteoid or immature bone within soft tissues, without direct attachment to the skeletal system [1]. It accounts for approximately 1% of all soft tissue sarcomas and 2–4% of all osteosarcomas. In contrast to conventional osteosarcoma of bone, ESOS typically occurs in adults, with a peak incidence in the fifth to sixth decades of life [2]. ESOS most commonly arises in the deep soft tissues of the extremities, retroperitoneum, or trunk. Superficial or subcutaneous involvement is distinctly uncommon and represents only a small minority of reported cases [3]. Because superficial lesions may clinically resemble benign soft tissue masses, delayed presentation and initial misdiagnosis are not unusual [4]. Radiologic findings are often nonspecific, and definitive diagnosis relies on histopathologic identification of osteoid produced by atypical tumor cells.

Due to its rarity, optimal treatment strategies for this condition remain controversial, particularly regarding the role of adjuvant chemotherapy and radiotherapy [5]. Management primarily involves wide surgical excision, ideally with negative margins. However, prognosis is generally unfavorable, with substantial risks of local recurrence and distant metastasis.

Here, we report a case of subcutaneous ESOS arising in the gluteal region of a middle-aged man, treated at a tertiary referral hospital in Samoa. This case highlights both the diagnostic challenges of superficially located ESOS and the practical constraints encountered in resource-limited healthcare settings.

## Case presentation

A 45-year-old Samoan man presented with a one-year history of a progressively enlarging, painless mass in the left gluteal region. The patient initially noticed a small, asymptomatic subcutaneous nodule, which gradually enlarged over the course of one year to approximately 10 cm in diameter. He denied fever, weight loss, trauma, infection, or other constitutional symptoms. There was no significant past medical history.

On physical examination, a firm, non-tender, poorly mobile subcutaneous mass with well-defined margins was observed. The overlying skin was intact, without erythema, ulceration, or other signs of inflammation.

**Fig. 1:**
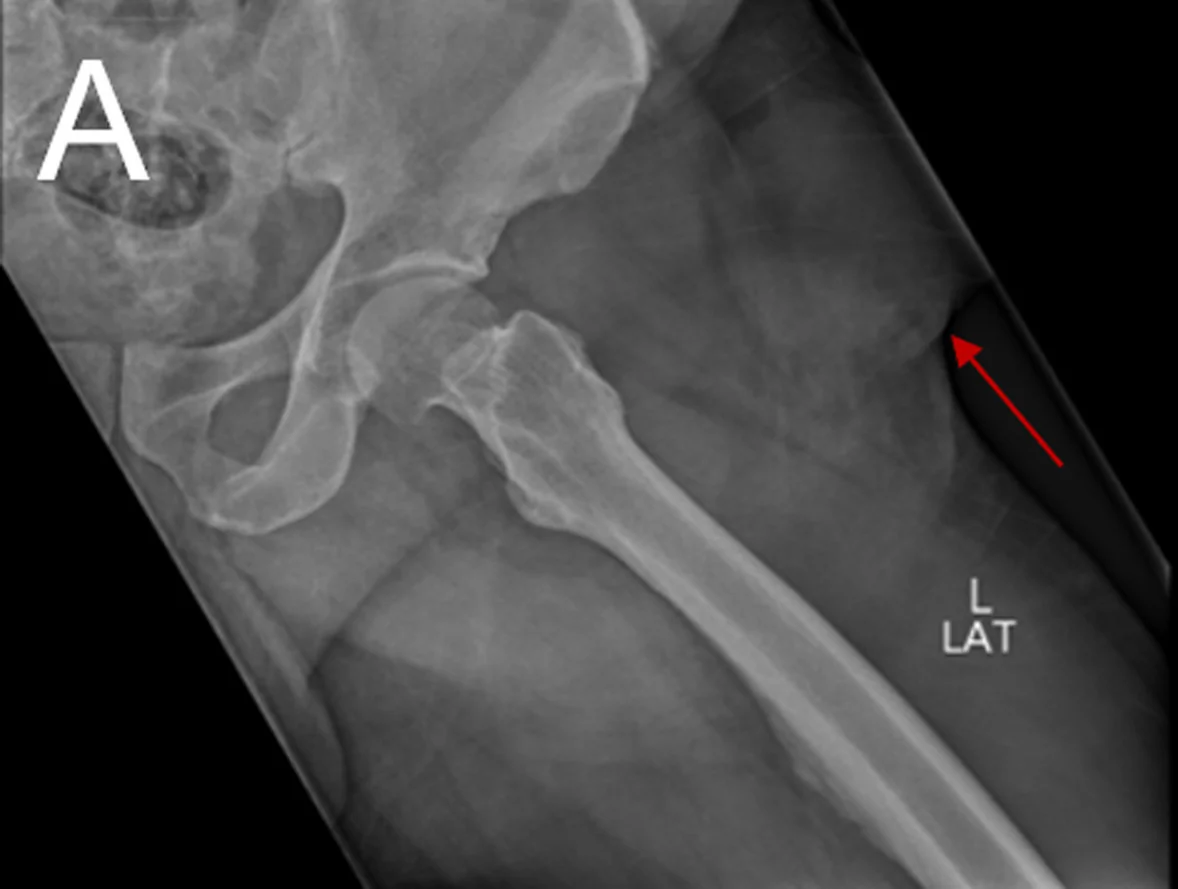
Pelvic radiograph demonstrating a soft tissue mass in the left gluteal region without calcification or underlying osseous involvement

Pelvic radiography demonstrated a well-circumscribed soft tissue mass in the left gluteal subcutaneous tissue measuring approximately 10 × 9 cm, without evidence of underlying bone involvement (Fig. 1).

**Fig. 2:**
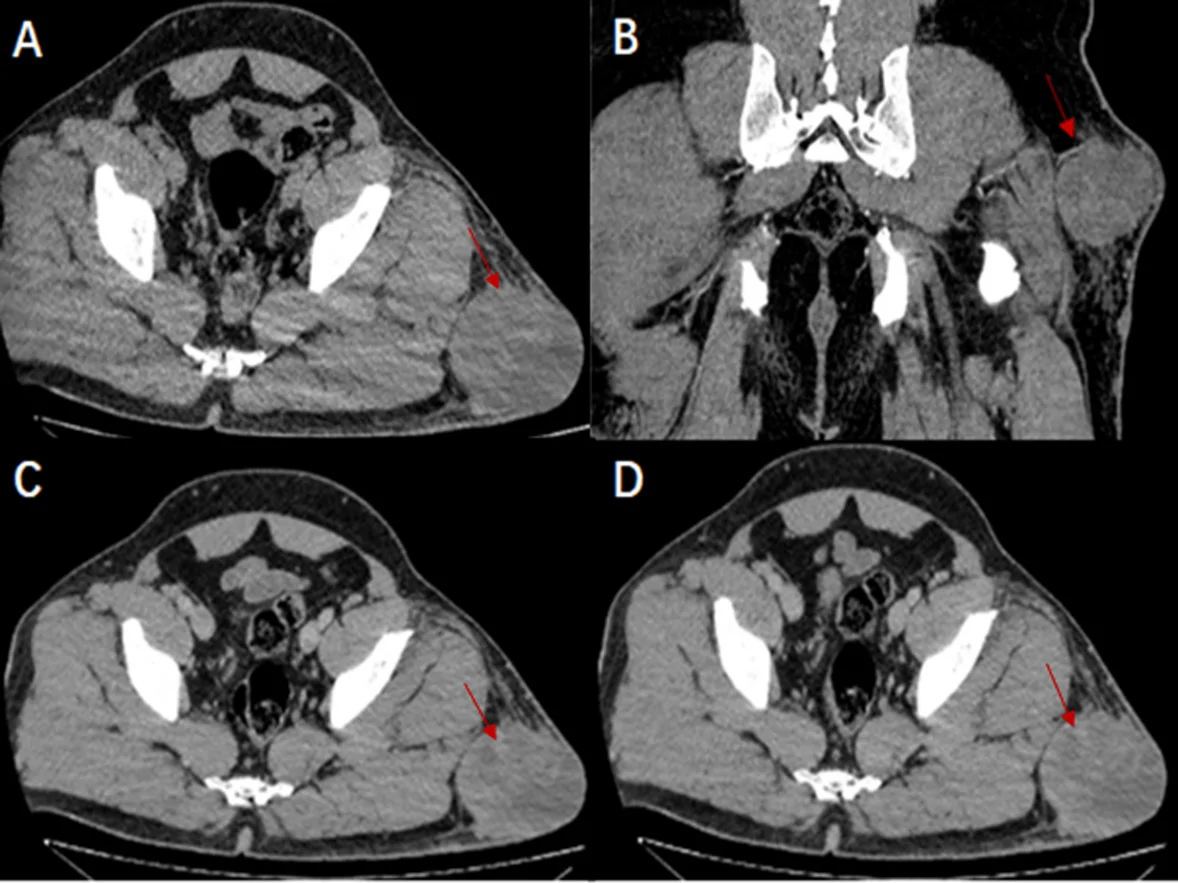
Contrast-enhanced CT of the pelvis demonstrating a large subcutaneous mass in the left gluteal region. **A** Axial non-contrast CT showing a well-defined soft tissue lesion. **B** Coronal reconstruction demonstrating the superficial location of the tumor (arrow) and its separation from the underlying bone. **C** Axial contrast-enhanced image showing heterogeneous enhancement. **D** Delayed-phase CT demonstrating mild to moderate delayed enhancement

Contrast-enhanced computed tomography (CT) revealed a large, well-defined subcutaneous lesion measuring 9.1 × 10.4 × 8.6 cm. On non-contrast CT, the lesion exhibited heterogeneous attenuation ranging from 14 to 40 Hounsfield units. Post-contrast imaging showed mild to moderate delayed enhancement, with arterial supply originating from branches of the left inferior gluteal artery. A small amount of perilesional soft tissue edema was noted, and the left gluteus maximus muscle was mildly compressed. No radiologic evidence of invasion into adjacent tissues or bone was observed (Fig. 2). Baseline chest CT was not performed, and no documented baseline chest radiography was available in the medical records.

Preoperative core needle biopsy was not performed. Although core needle biopsy is generally recommended for large or suspicious soft tissue masses before definitive surgery, the lesion was clinically superficial, well circumscribed, and considered resectable. In addition, access to biopsy needles and specialized sarcoma diagnostic pathways was limited in the local setting.

**Fig. 3:**
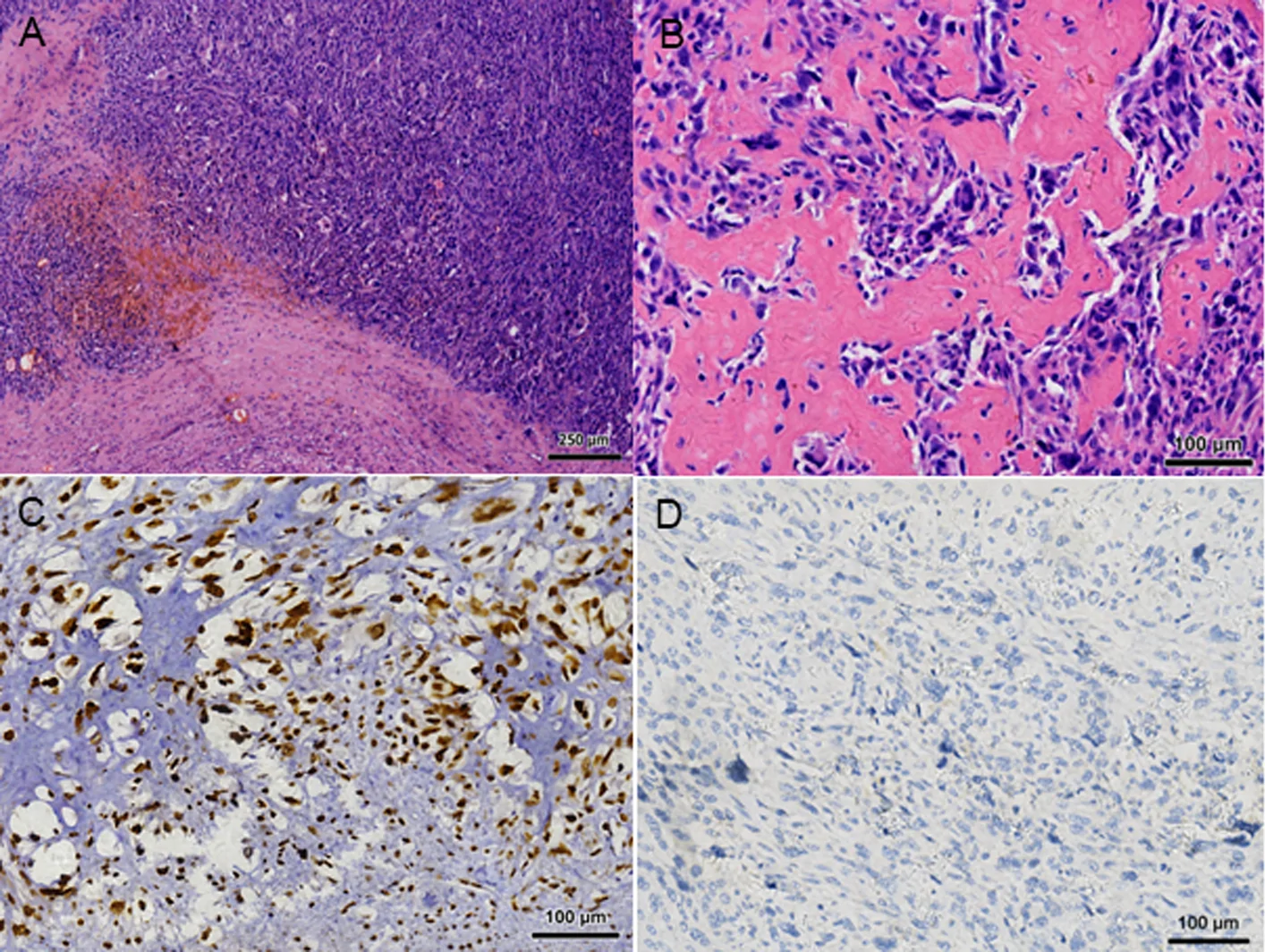
Histopathological and immunohistochemical features of extraskeletal osteosarcoma. **A** Low-power H&E image showing a spindle to pleomorphic high-grade sarcoma with neoplastic osteoid formation (original magnification × 100; scale bar, 250 μm). **B** High-power H&E image demonstrating marked cytologic atypia, nuclear pleomorphism, and osteoid produced by atypical tumor cells (original magnification × 200; scale bar, 100 μm). **C** SATB2 immunohistochemistry showing diffuse nuclear positivity in tumor cells, supporting osteoblastic differentiation (original magnification × 200; scale bar, 100 μm). **D** Pan-cytokeratin immunostaining is negative in tumor cells, helping exclude epithelial differentiation and making synovial sarcoma less likely (original magnification × 200; scale bar, 100 μm)

The patient subsequently underwent wide local excision with curative intent. Intraoperatively, the tumor appeared encapsulated and was not adherent to surrounding structures. No gross residual disease was observed intraoperatively. Histopathological examination demonstrated a spindle to pleomorphic high-grade sarcoma with marked cytologic atypia and abundant osteoid matrix produced by atypical tumor cells (Fig. 3). Low-power histologic examination revealed infiltrative tumor growth with irregular eosinophilic osteoid matrix deposition surrounded by atypical spindle and pleomorphic tumor cells. High-power examination demonstrated large markedly atypical pleomorphic cells with prominent nuclei, moderate eosinophilic cytoplasm, nuclear pleomorphism, and frequent mitoses, including atypical forms.

Immunohistochemical staining demonstrated diffuse nuclear positivity for SATB2, supporting osteoblastic differentiation, while tumor cells were negative for S100, SOX10, pan-cytokeratin, and ERG. Representative SATB2 and pan-cytokeratin immunostains are shown in Fig. 3. The overall morphologic, immunophenotypic, and radiologic findings supported the diagnosis of subcutaneous extraskeletal osteosarcoma. Microscopic examination showed that the tumor extended into the deep resection margin, consistent with a positive microscopic margin (R1 resection). The closest margin distance was not specified in the pathology report. The tumor was described as high grade. Frequent mitoses, including atypical forms, were reported; however, a formal mitotic count and FNCLCC grade were not specified in the available pathology report. The patient declined adjuvant therapy after surgery and was subsequently lost to follow-up.

## Discussion

Extraskeletal osteosarcoma (ESOS) is an uncommon and aggressive soft tissue sarcoma, representing approximately 1% of all soft tissue sarcomas. Subcutaneous ESOS is particularly rare, accounting for a small minority of reported cases [6]. Because superficial tumors often present as slow-growing, painless masses, they may clinically resemble benign entities such as lipoma, epidermal cyst, or organized hematoma [4, 7, 8]. This deceptively indolent presentation frequently contributes to delayed evaluation, as observed in our patient, who sought medical attention only after the lesion had reached a substantial size.

Radiologic findings in ESOS are variable and often nonspecific. Calcification or ossification has been reported in up to half of cases; however, the absence of mineralization does not exclude the diagnosis [7, 8]. In our case, no obvious calcification was detected on radiography or CT imaging, which further complicated preoperative suspicion. Contrast-enhanced CT demonstrated a well-circumscribed subcutaneous mass with mild to moderate delayed enhancement and no bone involvement. Although magnetic resonance imaging (MRI) is generally preferred for soft tissue tumor characterization, CT remains a pragmatic and accessible modality in many resource-limited settings. In this context, CT provided essential information regarding lesion extent, vascular supply, and the absence of skeletal invasion, thereby facilitating surgical planning [9].

Definitive diagnosis of ESOS relies on histopathological identification of osteoid produced directly by atypical mesenchymal tumor cells. In the present case, the tumor demonstrated spindle to pleomorphic high-grade morphology, marked cytologic atypia, frequent mitoses including atypical forms, and neoplastic osteoid formation. SATB2 positivity supported osteoblastic differentiation, while negative staining for S100, SOX10, pan-cytokeratin, and ERG helped exclude several morphologic mimics [10, 11].

The main differential diagnoses include myositis ossificans, dedifferentiated liposarcoma with osseous metaplasia, malignant peripheral nerve sheath tumor, synovial sarcoma, and other high-grade spindle cell sarcomas with heterologous differentiation [12]. Myositis ossificans was considered unlikely because the lesion lacked a zonal maturation pattern and instead showed osteoid matrix intimately associated with atypical pleomorphic tumor cells. Dedifferentiated liposarcoma with osseous metaplasia was not favored because no well-differentiated lipomatous component or adipocytic atypia was identified. Malignant peripheral nerve sheath tumor and melanoma were considered less likely because tumor cells were negative for S100 and SOX10. Synovial sarcoma and sarcomatoid carcinoma were also unlikely given the absence of epithelial differentiation and negative pan-cytokeratin staining. ERG negativity additionally argued against vascular sarcoma.

Preoperative core needle biopsy is generally recommended for large or suspicious soft tissue masses before definitive surgery, particularly when sarcoma is within the differential diagnosis. In this case, preoperative biopsy was not performed. This reflected both clinical and resource-related circumstances. The lesion was superficial, well circumscribed, and considered surgically resectable on preoperative evaluation. In addition, access to biopsy needles was limited, with supplies depending largely on external donation, and specialized sarcoma diagnostic pathways were not well established locally. Before radiology support provided by the Chinese medical team, CT-based evaluation for soft tissue tumors was not routinely integrated into local diagnostic pathways. This case therefore highlights the importance of strengthening imaging-based assessment, biopsy pathways, and multidisciplinary awareness for soft tissue tumors in Samoa.

Wide surgical excision with negative margins remains the cornerstone of treatment for localized ESOS [6]. In our patient, no gross residual disease was observed intraoperatively; however, microscopic examination later showed involvement of the deep resection margin, consistent with R1 resection. The closest margin distance was not specified in the pathology report. The role of adjuvant chemotherapy and radiotherapy in ESOS remains controversial [10]. Unlike conventional osteosarcoma of bone, ESOS appears to be less responsive to standard osteosarcoma chemotherapy regimens. Retrospective studies have reported conflicting results, with some suggesting limited survival benefit from chemotherapy [5, 13], while others indicate possible improvements in local control or disease-free survival in selected patients [3, 13]. Similarly, radiotherapy may enhance local control in large tumors or in cases with close or positive margins, although ESOS is traditionally considered relatively radioresistant [12, 14]. Given the absence of randomized prospective data, treatment decisions should therefore be individualized, balancing tumor size, margin status, patient factors, and available resources.

The prognosis of ESOS is generally poor, with reported local recurrence and distant metastasis, most commonly to the lungs, occurring in a substantial proportion of patients [3, 14]. Tumor size greater than 5 cm, age over 45 years, and positive surgical margins have been associated with worse outcomes [3]. While superficially located tumors may offer a better prognosis due to earlier detection [15, 16], the patient in this case presented with a large lesion measuring over 10 cm and had microscopic deep margin involvement, both of which are adverse prognostic factors. The patient declined adjuvant therapy and was subsequently lost to follow-up, precluding assessment of long-term outcome.

Several limitations should be acknowledged. Preoperative core needle biopsy, which is generally recommended for large or suspicious soft tissue masses, was not performed because the lesion was clinically superficial, well circumscribed, and considered resectable in a resource-limited setting where access to biopsy needles and specialized sarcoma diagnostic pathways was limited. The absence of documented baseline chest staging limited assessment of pulmonary metastasis at diagnosis. Microscopic examination showed involvement of the deep resection margin, consistent with R1 resection, but the closest margin distance was not specified. Frequent mitoses, including atypical forms, were described; however, formal mitotic count and FNCLCC grade were not provided. Gross specimen and intraoperative photographs were also unavailable.

Beyond these case-specific limitations, this report illustrates broader challenges in the diagnosis and management of rare sarcomas in resource-limited healthcare systems. Limited access to advanced imaging, biopsy supplies, subspecialty pathology services, multimodal oncologic therapy, and long-term surveillance may affect diagnostic pathways, treatment decisions, and outcome assessment. This case therefore underscores the importance of strengthening imaging-based evaluation, tissue diagnosis pathways, multidisciplinary collaboration, and continuity of oncologic care in similar settings.

## Conclusion

Subcutaneous extraskeletal osteosarcoma is an exceptionally rare and aggressive soft tissue malignancy that may initially present with deceptively benign clinical features. This case demonstrates that even superficially located, slow-growing masses warrant careful imaging evaluation, timely tissue diagnosis when feasible, and comprehensive histopathological assessment. Pathological confirmation, including representative histologic examination and immunohistochemical analysis, is essential for establishing the diagnosis and excluding morphologic mimics. Surgical excision remains the mainstay of treatment, but margin status, pulmonary staging, and access to adjuvant therapy are critical considerations. Early recognition and strengthening of multidisciplinary diagnostic pathways are important to optimize management, particularly in healthcare systems with limited oncologic resources.

## Data Availability

All data supporting the findings of this study are included in the article. No additional datasets were generated or analyzed during the current study.
